# Microglia: Mediators of experience-driven corrective neuroplasticity

**DOI:** 10.1016/j.ibneur.2025.05.013

**Published:** 2025-06-05

**Authors:** Lara Rogerson-Wood, Atomu Sawatari, Catherine A. Leamey

**Affiliations:** School of Medical Sciences (Neuroscience theme), Faculty of Medicine and Health, University of Sydney, NSW 2006, Australia

**Keywords:** Environmental enrichment, Visual System, Microglia, Plasticity, Neurodevelopment, Ten-m3, Neurodevelopmental condition, Experience

## Abstract

Neural connectivity is essential for brain function: this is initially established via early axon guidance mechanisms and subsequently refined by synaptic pruning. Alterations in the patterns of neural connectivity, arising due to changes in either of these processes, are found in neurodevelopmental conditions. Microglia, the brain’s resident immune cell, are recognised mediators of synaptic pruning. Unlike axon guidance, synaptic pruning occurs over protracted periods of postnatal life and can be profoundly impacted by experience. Little is known about whether targeted microglial synaptic pruning could be recruited to compensate for alterations in neural connectivity arising due to deleterious changes in other neurodevelopmental processes, such as axon guidance. Here we review our recent work which has addressed this by examining the effect of Environmental Enrichment (EE) on the miswired visual circuitry of mice lacking the axon guidance molecule Ten-m3. Notably, exposure to EE commenced around birth (but not from weaning or later) triggered selective removal of miswired retinal inputs in the visual thalamus of these Ten-m3 knockout mice. Most importantly, our work identifies selective microglial engulfment of neural connections during a defined postnatal window, as a likely mediator of this effect of early EE. The findings reviewed here emphasise the importance of early life experience in shaping neural circuitry, particularly when early development has been compromised by genetic factors. They also provide a potential mechanistic underpinning for the results of recent clinical trials investigating the effectiveness of early, experience-based interventions for human neurodevelopmental conditions.

Microglia are the brain’s primary resident immune cell population (Prinz et al., 2019, Sierra et al., 2019). Over the last decade or so, substantial evidence has emerged indicating that microglia also have direct involvement in the removal of excess/unwanted synaptic connections in both developing and adult circuits (Wong and Favuzzi, 2023, Zhao et al., 2024). Despite recognition of this important aspect of microglial function, there is scant evidence as to whether and how microglial function might be recruited to help fix connectivity defects caused by deleterious changes to other developmental processes. Recent work from our lab has addressed this gap through use of the widely studied, Environmental Enrichment (EE) paradigm (Kempermann, 2019) and the visual system of mice lacking the axon guidance molecule Ten-m3. Here, we highlight our recent work demonstrating an intriguing capacity of EE during a defined window of postnatal development to drive selective removal of the most miswired retinal projections in the visual thalamus of these Ten-m3 knock-out (KO) mice (Eggins *et al.*, 2019). Most importantly, this work also identified microglia as a likely mediator of this EE-triggered ‘corrective’ pruning (Rogerson-Wood et al., 2022, Rogerson-Wood et al., 2024). These findings are discussed in the context of other recent findings regarding microglia, EE-driven plasticity, neural circuitry development and human neurodevelopmental conditions. Knowledge of the basic organising principles of adult murine visual circuitry, and the involvement of Ten-m3 in their establishment, are critical pre-requisites for understanding the significance of our findings and thus will also be presented (sections 1.1–1.2).

## Broad organisation of the adult mouse visual system

**Fig. 1:**
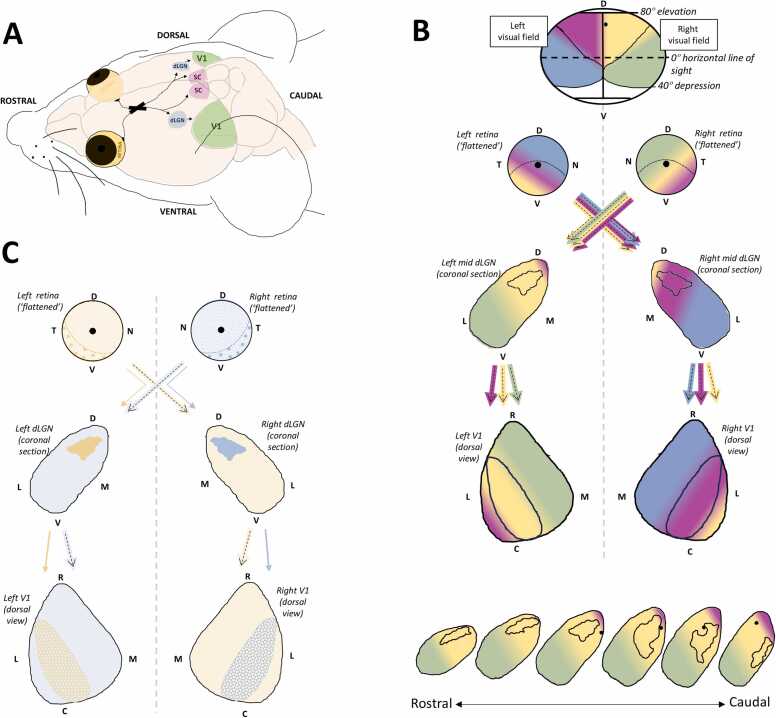
Schematic overview and breakdown of the broad organisation of the vision forming pathway in mice. A, In-situ schematic overview detailing the location of the retina (yellow), superior colliculus (SC - pink) and V1 (Green) in mice. At the midline, a large portion of RGCs decussate in the optic chiasm (OC – thick black cross in A) to form the contralateral RGC projection. The remainder form the ipsilateral RGC projection. RGC axons then pass through the optic tract (OT). Most RGC axons project to the SC, with a portion also sending collateral projections to the dLGN. B**,** Schematic overview of visuotopic organisation. The mouse visual field (displayed at top – coloured regions only) goes from roughly 40º below the horizontal, up to around 80º elevation and 120^º^ laterality. The binocular visual field (pink and yellow regions) is relatively small and very dorsally orientated. The visual receptivity of the retina, dLGN and V1 is colour coded with respect to the visual field schematic (top). the small black stars in 4 of the 6 coronal sections, are an example of a visuotopically corresponding projection line in series coronal sections - dLGN loci all receptive to the same point in visual space (see Ai – top, for rough point). C**,** schematic overview of eye specific projection patterns. Ipsilateral RGCs (left - dark yellow; right - dark blue) arise from a small, ventral temporal portion of the retina. Contralateral RGCs (light yellow - left, light blue - right) arise from the entire retina. In the dLGN, ipsilateral RGCs synapse in a small dorsal-medially located patch (dark yellow - left; dark blue -right), largely segregated from contralateral RGC inputs (light blue - left; light yellow - right). in V1, relayed ipsilateral retinal input from the dLGN synapses in a lateral segment (‘checked’ dark yellow – left V1; ‘checked’ dark blue – right V1) where it intermingles with visually corresponding contralateral retinal input. Grey dotted line (B**,** C) indicates midline. Retinal ganglion cell (RGC); dorsal lateral geniculate nucleus (dLGN); primary visual cortex (V1); SC (superior colliculus); VTC (ventral temporal crescent); ON (optic nerve); OT (optic tract); optic chiasm (OC); dorsal (D); ventral (V); nasal (N); temporal (T); lateral (L); medial (M); rostral (R); caudal (C). *schematic - not to scale*. See main text for literature references.

Like other mammals (including humans), mice possess a visual system where a central portion of their visual field can be seen by both eyes (termed the ‘binocular’ visual field). Due to the orientation of the eyes and prominent snout [see Fig. 1A; (Lieberman *et al.*, 2008)], this binocular visual field is relatively small (e.g., compared to humans) and more dorsally orientated (Fig. 1B – pink and yellow). Each retina receives light mostly from the same-side visual field [(Johnson *et al.*, 2021); Fig. 1B], with only a very small [∼22 % of total retina area; (Drager and Olsen, 1980)] portion [ventrotemporal crescent (VTC); Fig. 1C] receiving input from the opposite-side visual field [(Johnson *et al.*, 2021); Fig. 1B)]. To facilitate downstream integration of corresponding alternate eye inputs, each retina has both crossed (contralateral) and uncrossed (ipsilateral) retinal ganglion cell (RGC) projections [(Drager and Olsen, 1980); Fig. 1C]. In mice, the latter constitutes only 2–3 % of the entire RGC population (Martersteck et al., 2017, Murcia-Belmonte and Erskine, 2019). Ipsilateral RGCs originate exclusively from within the retinal VTC, whereas contralateral RGCs originate from throughout the retina (Fig. 1C). Even within the VTC, ipsilaterally projecting RGC populations constitute only around 15 % (Fig. 1C), with the remaining RGC axons projecting contralaterally (Drager and Olsen, 1980, Coleman et al., 2009, Sterratt et al., 2013; Rompani et al., 2017).

One of the main retinorecipient structures in mammals is the dorsolateral geniculate nucleus (dLGN). In mice, 30–40 % of all RGCs synapse here (Guido, 2018). Due to the decussation pattern of RGCs in mice (i.e., only 2–3 % of RGCs project ipsilaterally), each dLGN receives visual input mostly from the opposite-side visual field, with only extreme dorsomediocaudal loci receiving input from the same-side visual field (Fig. 1B). Contralateral and ipsilateral RGC inputs to the dLGN remain largely segregated (Fig. 1C), with only a small degree of binocular convergence occurring in more dorsal dLGN regions (Howarth et al., 2014, Jaepel et al., 2017, Rompani et al., 2017, Sommeijer et al., 2017, Huh et al., 2020, Bauer et al., 2021). Contralateral RGC axons terminate throughout the dLGN, only absent from a small (Guido, 2018), irregular shaped patch (Fig. 1C) constituting the termination zone for ipsilateral RGCs axons (Jaubert-Miazza *et al.*, 2005). This ipsilateral patch is very dorsolaterally located in the rostral dLGN (i.e., confined to the dorsal half of the dLGN), but shifts slightly more ventromedially in more caudal loci [still largely confined to the dorsal two thirds of the dLGN; (Godement et al., 1980, Antonini et al., 1999, Coleman et al., 2009); Fig. 1B]. Arguing for its functional importance, this patch covers approximately 10–15 % (depending on the analysis approach) of the total dLGN coronal cross-sectional area – a relatively large area when we consider that ipsilateral RGCs only make up only ∼2–3 % of all RGCs projecting from the retina. Studies in rats (almost identical visual system to mice) have shown ipsilateral and contralateral RGC terminal zones in the dLGN (see Fig. 1B) each have their own separate visuotopic map (Reese, 1988), but these tend to be very visuotopically aligned [(Reese and Jeffery, 1983); Fig. 1B). As illustrated in Fig. 1B, dorsal to ventral movement in the mouse dLGN corresponds to progressively more inferior regions of the visual field, whereas medial to lateral directions in the nucleus correspond to increasing laterality in the visual field (Piscopo *et al.*, 2013).

**Fig. 2:**
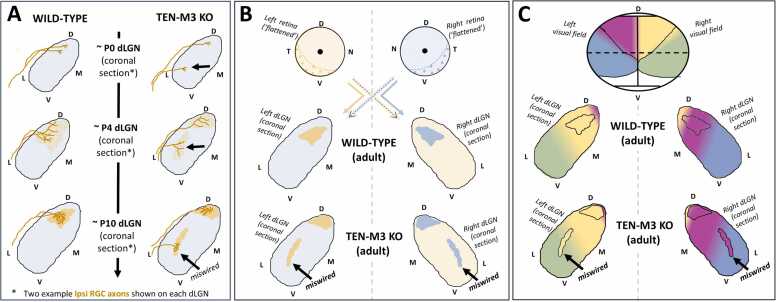
Ipsilateral RGC axons in Ten-m3 KO mice exit the optic tract early during early development such that they terminate in the adult dLGN in a significantly ventral-laterally elongated region, that is not visuo-topically aligned with contralateral RGC terminals. A, in both WT and Ten-m3 KO mice, ipsilateral RGC axons (overlayed over schematic dLGN sections in brown) grow ventral to dorsal in the optic tract along the dorsolateral dLGN border, exiting the optic tract between embryonic day (E)18 and birth (P0). in Ten-m3 KO mice though, a portion of these ipsilateral RGC axons exit the optic tract early to terminate significantly more ventral-laterally in the dLGN (black arrow), than they do in WT mice. The monocular segregation of ipsilateral (dark yellow) and contralateral (light blue) retinogeniculate terminal zones, still occurs normally in Ten-m3 KO mice. B, schematic comparing eye specific retinogeniculate projection patterns in the dLGN of Ten-m3 KO and WT mice - rather than synapsing in a dorsal-medially confined ‘patch’ (WT mouse patterning), ipsilateral retinogeniculate terminals in Ten-m3 KO mice synapse significantly more ventral-laterally (black arrow) and a little more dorsal-medially. C**,** schematic comparing visuotopy in the dLGN of Ten-m3 KO and WT mice. The visual receptivity of the retina and dLGN is colour coded - the binocular portion of the visual field (region seen by both eyes) is in pink (left visual field) and yellow (right visual field) and the monocular portions of the visual field, in blue (left visual field) and Green (right visual field). The altered innervation pattern of ipsilateral RGCs in the Ten-m3 KO mice (outlined in B), disturbs the visuotopic alignment of ipsilateral and contralateral inputs (black arrow) that is usually found in the dLGN of wildtype mice. A-C**,** schematic of mid-rostral, sections of dLGN are presented. Ventral-temporal crescent (VTC); Knock-out (KO); dorsal lateral geniculate nucleus (dLGN); dorsal (D); ventral (V); lateral (L); medial (M). *schematic - not to scale*. See main text for literature references.

The main projection target of dLGN relay neurons [the only dLGN neuron type which projects outside the dLGN – (Guido, 2018)] is the primary visual cortex [V1; (Bienkowski et al., 2019, Froudarakis et al., 2019)]. Thalamocortical axons relaying contralateral retinal input, terminate throughout V1 with a slightly higher density of terminals in the medial two thirds (Fig. 1B). Those relaying ipsilateral retinal input, terminate in the lateral third of V1 [Fig. 1A – bottom; (Dräger, 1975, van Beest et al., 2021)], often termed “the binocular zone” (Antonini, Fagiolini and Stryker, 1999). Again, a relatively large region when we consider that ipsilateral RGCs only make up only ∼2–3 % of all RGCs projecting from the retina. As in the dLGN, each hemisphere of V1 predominantly receives input from the opposite-side visual field, with a minor representation of the same side visual field (dorsomedial location) at extreme caudal-lateral loci (Kleinfeld et al., 2017, Song et al., 2020, van Beest et al., 2021); Fig. 1A]. As depicted in Fig. 2B, progressing from the midline to the lateral border of each V1 hemisphere, maps from the extreme peripheral visual field to the central visual field. Going from rostral to caudal in each V1 hemisphere, corresponds to increasing elevations in visual space (Dräger, 1975, Drager, 1978, Wagor et al., 1980, Song et al., 2020). Alternate eye inputs onto individual neurons within the ‘binocular zone’ of V1 generally have receptive fields that are mostly overlapping [average centre to centre separation of roughly 9.5º; (Merlin *et al.*, 2013)].

## Ten-m3 guides the initial developmental ingrowth trajectory and final adult positioning of ipsilateral RGC terminals in the mouse dLGN

To establish connections between two brain regions, axons must follow a highly specific growth path through the brain over early neurodevelopment to reach their target structure. For ipsilateral RGC axons terminating in the dLGN, this involves growth along the optic nerve, **not** crossing over at the optic chiasm, growth along the (ipsilateral) optic tract and then exiting (the optic tract) to enter and terminate in the dLGN (Herrera, Chédotal and Mason, 2024). The latter step occurs between ∼E18 and birth in mice. In mice, all RGCs grow in a ventral to dorsal direction along the optic tract which wraps around the lateral border of the dLGN (Pierre Godement, Salaün and Imbert, 1984; Pfeiffenberger et al., 2005; Glendining et al., 2017). Ipsilateral RGC axons, however, are unique in that they typically remain entirely confined to the optic tract until they reach the dorsal half of the dLGN [(Glendining *et al.*, 2017); Fig. 2A], their visuotopically appropriate terminal zone (Fig. 1B).

Studies have shown that the transmembrane glycoprotein Ten-m3 (Oohashi *et al.*, 1999), plays a key role in guiding this specific ipsilateral RGC in-growth trajectory to the dLGN. Ten-m3 is expressed in topographically corresponding gradients in the retina and dLGN during development i.e., high expression in retinal VTC and dorsal dLGN (Leamey and Sawatari, 2014, Leamey and Sawatari, 2019). In mice lacking Ten-m3 (aka Ten-m3 knock-out mice), ipsilaterally projecting RGC axons exit the optic tract early, such that they enter the dLGN far more ventrally than typically seen in wild-type (WT) mice [(Glendining *et al.*, 2017); depicted in Fig. 2A]. This altered in-growth trajectory translates into a highly consistent, elongation of their ipsilateral RGC terminal zone (Leamey et al., 2007a). This ‘elongation’ exists in both dorsomedial and ventrolateral directions, with the latter being the most apparent (Fig. 2B). The ventrolateral elongation becomes more pronounced when transitioning from caudal to more rostral dLGN (Leamey et al., 2007b). Because of this, the usual visuotopic alignment of alternate eye input to the dLGN (Fig. 1B) is profoundly disrupted in Ten-m3 KO mice. Of note, dLGN regions which usually receive visual input only from the peripheral visual field (purple and green regions in Fig. 2C), receive information from the central portion of the visual field (pink and yellow regions in Fig. 2C) in Ten-m3 KO mice (Fig. 2C).

Accompanying this aberrant anatomical ipsilateral retinogeniculate connectivity in Ten-m3 KO mice is altered visuotopic mapping of relayed ipsilateral eye input to V1 (Merlin *et al.*, 2013). Of note, ectopic ipsilateral retinal drive (relaying central visual field information) is present in medial V1 of Ten-m3 KO mice [(Merlin et al., 2013); medial V1 usually only receives contralateral eye input in mice (Fig. 1C)]. Further, individual and nearby cells in V1 (both in medial and lateral V1) receive alternate eye specific inputs that relay information from completely non-overlapping regions of the visual field. Retrograde tracing experiments from V1 have shown anatomically, that the patterning of dLGN relay neuron projections to V1, are not detectably different in Ten-m3 KO mice to that of WT mice (see Fig. 1C) (Leamey et al., 2007a). Thus, the ectopic mapping of ipsilateral eye input in V1 of Ten-m3 KO mice, appears to be a downstream transference of the earlier aberrant ipsilateral retinogeniculate wiring (described above), verses being an additional axon guidance defect affecting dLGN relay neurons projecting to V1.

It is important to note that loss of Ten-m3 in mice seems to leave topographical mapping of contralateral inputs to the dLGN and downstream V1, largely unchanged (Leamey et al., 2007b, Merlin et al., 2013). The distinctive spatial segregation of alternate eye inputs to the dLGN that usually develops between P4 and eye opening (P12–14) in mice (Godement et al., 1984, Jaubert-Miazza et al., 2005)], also appears largely unaffected by loss of Ten-m3 [Fig. 2A (Leamey et al., 2007a, Glendining et al., 2017)]. Further, no changes in the overall number, retinal origin (Fig. 2B), nor terminal area of ipsilateral RGCs (in the dLGN) have been observed in Ten-m3 KO mice (Leamey et al., 2007b). These latter findings indicate that Ten-m3 is likely not essential for guiding the earlier growth trajectory of ipsilateral RGCs axons through the optic nerve, chiasm, and tract [see (Herrera, Chédotal and Mason, 2024) for review], nor in determining ipsilateral RGC genesis and/or specification in the retina [see (Murcia-Belmonte and Erskine, 2019) for review]. Thus Ten-m3 appears to have a specialised role in setting up ipsilateral retinogeniculate connectivity. Consistent with the widely acknowledged importance of visuotopy and aligned binocular inputs to vision, binocular (but interestingly not monocular) visually-mediated behaviour is also severely compromised in Ten-m3 KO mice (Leamey et al., 2007a, Blok et al., 2020).

## Early EE drives removal of the most mis-wired ipsilateral retinogeniculate axon terminals in Ten-m3 KO mice

**Fig. 3:**
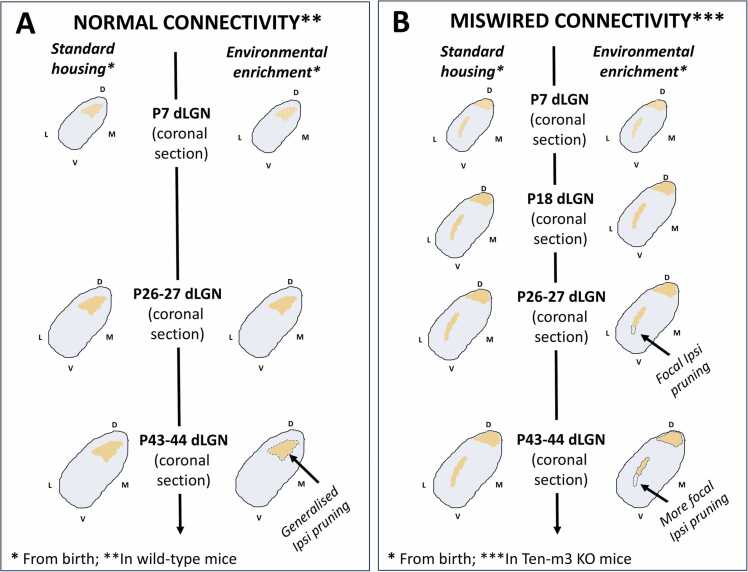
Environmental enrichment from **b**irth in Ten-m3 KO mice triggers focal pruning of their most visuo-topically mis-mapped ipsilateral retinogeniculate terminals, in a defined postnatal window. A, in wild-type mice, EE from **b**irth until P7 or P26–27 does not change the area occupied **b**y ipsilateral retinogeniculate terminals (dark yellow; the area where contralateral retinogeniculate terminals occupy is in light **b**lue). EE from **b**irth until P43–44 does trigger generalised pruning of ipsilateral retinogeniculate terminals though (I.e., not focal; delineated **b**y **b**lack dotted line). B, in Ten-m3 KO mice, EE from **b**irth until P7 or P18 also does not alter the area occupied **b**y ipsilateral retinogeniculate terminals (dark yellow). EE from **b**irth until P26–27 or P42–44, however, triggers very focal pruning (delineated with arrow and **b**lack dotted line) of their most visuotopically mis-mapped ipsilateral retinogeniculate terminals (I.e., those most ventral-laterally located in the dLGN). this EE-trigger pruning in Ten-m3 KO mice is more pronounced at P42–44 than at P26–27, suggesting it is actively underway at P26–27. Schematic of mid-rostral sections of dLGN are presented. Knock-out (KO); dorsal lateral geniculate nucleus (dLGN); dorsal (D); ventral (V); lateral (L); medial (M); postnatal day (P). see main text for literature references.

Environmental enrichment (EE) is a protocol that aims to increase cognitive, physical, and social engagement in laboratory rodents. It does this through use of substantially larger cages that typically contain more novel objects (changed regularly - this often includes a running wheel) and more mice. It is a long-studied protocol (Rosenzweig and Bennett, 1996), having been shown to have a wide array of typically, very beneficial effects on the brain [including on the normal development of the visual system (Bibollet-Bahena *et al.*, 2023); see (Kempermann, 2019) for broad review of EEs effects]. Of note, research from our lab has characterised an intriguing capacity of EE to trigger targeted removal of the most profoundly miswired ipsilateral retinal inputs in the dLGN of Ten-m3 KO mice (Eggins et al., 2019, Rogerson-Wood et al., 2022, Rogerson-Wood et al., 2024) (Fig. 3B). This anatomical effect of EE was accompanied by substantial improvements in a usually defective, visually mediated behaviour - the looming stimulus escape response (Eggins et al., 2019, Blok et al., 2020). Importantly, both the anatomical and behaviour effects of EE were developmentally restricted: only EE from birth (Fig. 3B), but not EE from P21 nor early adulthood (the latter two effects not shown in figure) was able to elicit these changes (Eggins et al., 2019; Blok et al., 2019). This temporal alignment of behaviour and anatomical effects strongly suggests that the EE-driven improvements in visually mediated behaviour in Tem-m3 KO mice, are at least in part, a consequence of the concurrent EE-driven removal of miswired ipsilateral retinogeniculate inputs to their dLGN [see (Blok *et al.*, 2020) for further discussion].

Curiously, EE from birth until P43–44 was able to trigger significant reductions in overall ipsilateral RGC terminal area in both the dLGN of wild-type (Fig. 3A) and Ten-m3 KO mice (Fig. 3B). In WT mice this effect was very small (Fig. 3A) however, appearing to merely reflect an acceleration of axonal refinement that also occurs in standard housed (SE) mice, but at a later age [see (Eggins *et al.*, 2019) for more discussion]. The timing of this effect also seems to align well with the last stage of developmental RGC axonal arbour pruning (occurring between ∼ P31 and P60) already characterised in the wild-type mouse dLGN (Noutel et al., 2011, Hong et al., 2014). The same EE regime in Ten-m3 KO mice, however (i.e., EE from birth until P43/44), drove additional pruning (Fig. 3) that did not occur in standard housed Ten-m3 KO mice, even by 5–8 months of age (Eggins *et al.*, 2019). Most importantly, this ‘extra’ pruning in the dLGN of Ten-m3 KO mice appeared highly directed towards the most profoundly visuo-topically miswired ipsilateral RGC axon terminals [i.e., those most ventral-laterally displaced - Fig. 3B; see Fig. 2C for overview of visuotopy; (Eggins *et al.*, 2019)].

Through analysis of additional age cohorts, we were able to more closely determine the exact developmental window of this EE -triggered ‘corrective’ pruning in Ten-m3 KO mice. EE-from birth did not change ipsilateral RGC terminal area nor distribution in their dLGN by P7 (Eggins *et al.*, 2019), nor by P18 (Rogerson-Wood *et al.*, 2024). This indicates that the pruning triggered by EE from birth in Ten-m3 KO mice, is likely not being facilitated by changes in the initial ingrowth of ipsilateral RGC terminals (occurring around birth – see section 1.2 for review), nor by co-opting the early developmental window of RGC axon arbour pruning that has been characterised in the dLGN [(occurring substantially between P5 and P10; (Godement et al., 1984, Jaubert-Miazza et al., 2005)]. Similar analyses undertaken in the dLGN of P26–27 Ten-m3 KO mice, however, did show significant reductions in ipsilateral RGC terminal area following EE from birth. Consistent with only the most visuotopically mismapped RGC inputs being removed, the same analyses in age matched (P26/P27) wild-type mice showed no pruning effect of EE from birth (Fig. 3A). Most importantly, the magnitude of the EE-triggered pruning effect in P26/27 Ten-m3 KO mice was significantly smaller than that found in P43/44 Ten-m3 KO mice [Fig. 3B; (Rogerson-Wood *et al.*, 2022)], indicating the EE-driven corrective pruning of the miswired ipsilateral RGC inputs is likely actively in progress by this earlier timepoint. Curiously, P26/P27 seems to not align with any known developmental bouts of structural RGC axon arbour pruning in the dLGN of wild-type mice.

## Evidence suggests microglia mediate the EE-driven corrective pruning in Ten-m3 KO mice

Given the many benefits EE can have on the nervous system [see (Kempermann, 2019) for a broader review], there has been growing interest in trying to understand the molecular/cellular mediators of these effects. Microglia are increasingly considered a potential candidate (Augusto-Oliveira and Verkhratsky, 2021, Chen et al., 2025). Microglia are a glial cell of the brain with a diverse array of functions. In one capacity they act as the brain’s main immune cell, behaving as any classic tissue resident macrophage by hoovering up dead/dying cells and responding to tissue injury as well as invading pathogens [see (Sierra et al., 2019, Paolicelli et al., 2022), for reviews]. There is also now a wealth of evidence showing they also help facilitate neural plasticity (the formation of new synapses and their removal) across both normal development and into adulthood [see (Durán Laforet and Schafer, 2024, Zhao et al., 2024) for reviews]. In wild-type mice for instance, pharmacological or genetic dampening of microglial function prevents the monocular segregation of alternate eye (RGC) inputs that occurs during early dLGN development [between P4 and eye opening; (Jaubert-Miazza et al., 2005, Schafer et al., 2012)]. Microglia also seem to mediate the last stage of developmental RGC axonal arbour refinement that occurs between roughly P31 and P60 in mice (Noutel et al., 2011, Hong et al., 2014), with microglial lysosomes in the dLGN containing substantially more RGC terminal fragments at P40 than at P30, P50, or P60 (Schafer *et al.*, 2016).

Most studies investigating microglia as a candidate cellular mediator of EE, however, have undertaken experiments orientated towards investigating their more immune-related functions [i.e., in animal models involving some form of overt immune challenge [see (Augusto-Oliveira and Verkhratsky, 2021) for review)]. Despite microglia’s recognised involvement in plasticity [(Durán Laforet and Schafer, 2024; Zhao, Umpierre and Wu, 2024)], relatively little work has been done into how EE might influence this aspect of their function.

Most of the research that has been done, has focused on the healthy adult rodent hippocampus - a brain region critical for the acquisition and consolidation of autobiographical memories (Lisman *et al.*, 2017) and an area of the adult brain with particularly elevated neuroplasticity levels. In the dentate gyrus (DG) of this structure, EE reliably triggers neurogenesis [including survival and integration of the newborn neurons; (Kempermann et al., 1997, Ehninger and Kempermann, 2003, Ziv et al., 2006, Reshef et al., 2014, Frechou et al., 2024, Barde et al., 2025) as well as concurrent increases in microglial density [only when exposed to EE for more than one month; (Ziv et al., 2006, Williamson et al., 2012, Reshef et al., 2014, Ziegler-Waldkirch et al., 2017, Singhal et al., 2021)] and morphological complexity (Xu et al., 2016, Ali et al., 2019, de Oliveira et al., 2020, Socodato et al., 2023). The latter result has also been seen in the dorsal hippocampus (Socodato *et al.*, 2023), a functional hippocampal subdivision particularly involved in spatial memory (Strange *et al.*, 2014). EE in this structure has further been shown to trigger increases in microglial interactions with, and uptake of, post-synaptic elements [i.e., PSD-95; (Socodato *et al.*, 2023)]. Finally, direct disruption of microglial function has also been shown to hinder EEs capacity to trigger neurogenesis [in the dentate gyrus; (Ziv et al., 2006, Reshef et al., 2014)] and improve spatial working memory (Reshef et al., 2014, Socodato et al., 2023). Note, EE-driven neurogenesis in the DG has been causally linked with the concurrent EE-driven improvements in spatial working memory (Frechou *et al.*, 2024).

Though these results do support a causal role of microglia in EE-driven hippocampal plasticity in the healthy brain, they do not shed light into how EE might influence this aspect of microglia function following disruptions to the processes of early neural circuit assembly, such as axon guidance. Errors in the axon guidance are thought to contribute (McFadden and Minshew, 2013, Van Battum et al., 2015, Canali et al., 2018) to at least some of the pronounced connectivity differences observed in human neurodevelopmental conditions (Fu et al., 2019, Carroll et al., 2021, Takeguchi et al., 2022, Xie et al., 2023). This line of inquiry is of potentially great therapeutic relevance, given the recently demonstrated effectiveness of early experience-based interventions in the treatment of human neurodevelopmental conditions (Woo and Leon, 2013, Woo et al., 2015, Aronoff et al., 2016, Whitehouse et al., 2021).

In response to this, our lab sought to investigate if microglia were involved in mediating the EE-triggered removal of miswired ipsilateral RGC axon terminals, that we had already characterised in the dLGN of developing Ten-m3 KO mice. In support of this idea, EE from birth was found to elicit highly localised microglial reactivity at the corrective pruning of P25 Ten-m3 KO mice (see Fig. 3B for overview of site in the dLGN), with no comparable effect in age-matched WT mice. High resolution analysis of these reactive microglia showed them to have an increased density at this locus, significant morphological changes (i.e., more amoeboid in shape; less ramified) and substantially increased lysosomal CD68 content (Rogerson-Wood *et al.*, 2022) - all hallmarks of microglia that are actively partaking in facilitating neuroplastic changes (Schafer *et al.*, 2012). This localised microglial reactivity followed a clearly defined time course - commencing between P18 and P21, peaking at P25 and ceased by P30 (as assessed via CD68 distribution in the dLGN) - and was accompanied by clear evidence of EE-driven targeted microglial engulfment of the most miswired ipsilateral RGC terminals (see Fig. 2 for overview of most miswired terminals) at P25, but not P18 (Rogerson-Wood *et al.*, 2024). Critically, these effects of EE on microglia were temporarily aligned with the developmental time-course of the EE-driven anatomical corrective pruning that we previously characterised in Ten-m3 KO mice (see Fig. 3B), strongly suggesting a causal role of microglia in the process.

Interestingly, the timing of this effect of EE on microglia in Ten-m3 KO mice [i.e., localised reactivity starting between P18 and P21, and finished by P30; (Rogerson-Wood *et al.*, 2024)], does not clearly align with any known bout of microglial-mediated retinogeniculate axon arbour pruning in the wildtype mouse dLGN. It does align with a developmental window (between ∼P18 and P30) of increased visual sensitivity for the retinogeniculate synapse (Hooks and Chen, 2006, Hooks and Chen, 2008, Lin et al., 2014, Tschetter et al., 2018), suggesting a role of vision in instructing the corrective pruning (this interpretation aligns with the observation that only the most visuo-topically mismapped RGCs in the Ten-m3 KO dLGN get removed). Anatomically changes during this window of neurodevelopment though, typically only involve an organisational reshuffle of axonal boutons on a maintained, broad axon scaffold (Hong *et al.*, 2014). Substantial microglial-mediated retinogeniculate axon arbour pruning in the dLGN (like what we see in Ten-m3 KO mice – Fig. 3B), has only been shown to occur in wildtype mice after the closure of this window of visual sensitivity (Noutel et al., 2011, Hong et al., 2014) or during earlier monocular segregation [occurring between P4 and P14; (Jaubert-Miazza et al., 2005, Dhande et al., 2011)]. Thus, despite being highly developmentally restricted, this effect of EE on the Ten-m3 KO dLGN appears somewhat distinct from the normal processes of retinogeniculate synapse development - this presents an interesting opportunity to probe novel molecular mechanisms of EE-driven microglial-mediated-plasticity. Though more work is certainly needed, previous studies provide precedence for the involvement of novel epigenetic drivers of neuroplasticity, that can be uniquely switched-on by early EE (Li et al., 2006, Arai et al., 2009, Baroncelli et al., 2016).

Very recently, the relative importance of microglia during normal neurodevelopment has recently been called into question. Current studies have shown that when microglia are genetically depleted throughout life in mice [‘FIRE’ mice; see (Rojo *et al.*, 2019)], neural circuitry and glial cell profiles develop and mature remarkably normally (Munro et al., 2024, Surala et al., 2024, O’Keeffe et al., 2025). Part of these findings included a demonstration that there is no detectable deficit in the early monocular segregation of alternate eye inputs in the dLGN of these mice (O’Keeffe *et al.*, 2025). Given these findings, as well as other studies showing astrocytes (Chung et al., 2013, Lee et al., 2022, Park and Chung, 2023, Kim et al., 2025) and oligodendrocyte precursor cells [OPCs; (Auguste et al., 2022, Buchanan et al., 2022)] can perform similar plasticity-related functions (i.e., engulfment of synaptic components), it appears highly likely that microglia have a degree of functional redundancy in the healthy developing brain.

Our work with Ten-m3 KO mice and EE reviewed here (Eggins et al., 2019, Rogerson-Wood et al., 2022, Rogerson-Wood et al., 2024), warrant caution in the over-interpretation of these findings, however. Based on our work, the plasticity-related demands placed on microglia during healthy neurodevelopment appear to be less than those placed on microglia in neural systems where there are also deleterious changes to other neurodevelopmental processes (in this case, axon guidance). Thus, while it is possible that the brain might appear developmentally unaffected by the lack of microglia when neurodevelopment is otherwise normal (Munro et al., 2024, Surala et al., 2024, O’Keeffe et al., 2025), this might not be the case when developmental defects (such as axonal guidance) are present. Similarly, while neural circuitry may develop adequately in wild-type mice raised in the arguably “impoverished” conditions of standard laboratory housing, our findings indicated this appears to be insufficient to drive the plasticity needed to correct aberrant wiring caused by axonal guidance errors. In this instance, enhanced levels of experience seem necessary to promote normalisation of neural connectivity and function. It is possible that this experience-driven corrective process may be critically dependent on having a degree of redundancy in the system and thus, the presence of fully functioning microglia. Further experiments, potentially using FIRE mice (Rojo *et al.*, 2019) that have been genetically engineered to also present with a defined and easily measurable axon guidance defect (such as what we see in Ten-m3 KO mice; Fig. 2), would help assess these possibilities.

## Implications for other models of neurodevelopmental miswiring and human conditions

There is interest in the idea that differences in the synaptic pruning actions of microglia during neurodevelopment (both over and under pruning), might be a primary deficit in some neurodevelopmental conditions (Mordelt and de Witte, 2023, Lukens and Eyo, 2024). This hypothesis is based primarily on the work showing that microglial engulfment of synaptic elements is a part of normal neurodevelopment [see (Frost and Schafer, 2016, Durán Laforet and Schafer, 2024) for review], as well as evidence of aberrant microglial activation and engulfment levels in human neurodevelopmental conditions (Vargas et al., 2005, Morgan et al., 2010, Tetreault et al., 2012, Suzuki et al., 2013). Though microglial dysfunction might indeed be a primary deficit in some neurodevelopmental conditions, our findings with EE and Ten-m3 KO mice (Eggins et al., 2019, Rogerson-Wood et al., 2022, Rogerson-Wood et al., 2024) combined with the recent results with FIRE [discussed above; (Munro et al., 2024, Surala et al., 2024, O’Keeffe et al., 2025)], posit an alternative interpretation: evidence for aberrant microglial reactivity and elevated microglia engulfment (of synaptic material) detected in neurodevelopmental conditions, might also be an critical compensatory event to help correct for axonal guidance errors. Thus, caution should be taken when developing therapies for neurodevelopmental conditions that aim to dampen microglial function over development.

To the best of our knowledge, our findings with EE and Ten-m3 KO mice that we review here, are the first to suggest microglia can help to correct a neural connectivity defect caused by an earlier axon guidance error. Interestingly, over-exuberant and/or altered patterns of connectivity have been reported in various human neurodevelopmental conditions (Bertero et al., 2018, Takeguchi et al., 2022, Openshaw et al., 2023, Xie et al., 2023) and associated rodent models (Bertero et al., 2018; Smith et al., 2019; Jain et al., 2020; Kennedy et al., 2020; Carroll et al., 2021). Further, evidence also exists for the aetiological involvement of axonal guidance and pathfinding errors in various neurodevelopmental disorders, including Autism (McFadden and Minshew, 2013, Van Battum et al., 2015, Canali et al., 2018). Thus, it is conceivable that the capacity of microglia to facilitate normative change in (developmentally) miswired circuitry, as discussed here in enriched Ten-m3 KO mice, could be of enormous therapeutic benefit in some human neurodevelopmental conditions. Moreover, we can also begin to understand why separate genetic variants impacting microglial function and early axon guidance, might not produce substantial developmental deficits in isolation (given the right environmental conditions), but manifest as a severe functional deficit when combined. Future work using EE determining if the EE-induced corrective microglial pruning observed in our work with Ten-m3 KO mice, generalises to promote correction of aberrant neural wiring in other brain systems and/or more established neurodevelopmental models, will be required to investigate these possibilities. Possible models to peruse include the 16p11.2 (Bertero et al., 2018, Li et al., 2021, Openshaw et al., 2023) and CNTNAP2 (Peñagarikano et al., 2015; Dawson et al., 2023) mouse models of autism, alterations in brain wiring and microglial pruning having been reported in both.

A striking feature of our findings with Ten-m3 KO mice, is the high degree of temporal specificity of the effect of EE on microglia (and anatomical corrective pruning) in the dLGN - the localised microglial reactivity, engulfment and anatomical pruning (Fig. 3) are all highly temporally restricted to a distinct window of development. This suggests that there is a developmental ‘critical period’ of sorts, during which EE enables microglia to identify and specifically prune miswired inputs. Broadly speaking, this is consistent with recent clinical trials reporting beneficial changes for children at risk of, or diagnosed with, Autism [a neurodevelopmental condition of heterogeneous origins: (Carroll *et al.*, 2021)] following interventions which enhance early life experience (Woo and Leon, 2013, Woo et al., 2015, Aronoff et al., 2016, Whitehouse et al., 2021). Moreover, there are examples of studies showing greater efficacy when these interventions commence earlier, verses later in development (Guthrie *et al.*, 2023). There remains though, an ongoing discussion/debate as to whether the available evidence conclusively points to an optimal ‘early’ timing for such experience-based interventions [e.g., (Towle *et al.*, 2020)]. Our results with Ten-m3 KO mice and EE, provide a clear mechanistic-level justification to establish key timepoints in the implementation of such experience-based interventions for human neurodevelopmental conditions involving overt (congenital – present from birth) neural connectivity defects.

## Concluding remarks

This article has reviewed recent evidence showing that EE can trigger selective microglial engulfment of miswired neural connectivity during a defined postnatal window, to compensate for an earlier developmental axonal guidance defect (Eggins et al., 2019, Rogerson-Wood et al., 2022, Rogerson-Wood et al., 2024). This discovery was enabled via examination of the highly stereotyped miswiring of ipsilateral RGC axon terminals, present in visual thalamus of Ten-m3 KO mice (Leamey et al., 2007b, Leamey and Sawatari, 2019). These findings are broadly consistent with existing literature showing that microglia mediate neuroplasticity, and EE can influence microglial functioning and enhance plasticity in the brain (Kempermann, 2019). They appear to be the first, however, to give direct evidence indicating microglia can aid in correcting neural connectivity defects caused by an axonal guidance defect and further, that this can be driven by EE. The research discussed here highlights the multi-faceted roles of microglia in neural plasticity and how pertinent early life experience is, in shaping the ultimate outcome of genetically compromised neurodevelopment. They point towards new directions for the development of therapeutic strategies.

## Funding statement

Lara Rogerson-Wood was in receipt of an Australian Government Research. Training Program Scholarship (full time from 1/1/23–30/9/23) for the initial rough drafting of this article. No other external funding was received by the authors for the creation of this article.

## CRediT authorship contribution statement

**Atomu Sawatari:** Conceptualization, Writing – review & editing. **Catherine A. Leamey:** Writing – review & editing, Supervision, Conceptualization. **Rogerson-Wood Lara Emily:** Writing – review & editing, Writing – original draft, Visualization, Investigation, Conceptualization.

## Declaration of generative AI and AI-assisted technologies in the writing process

AI was not used in the creation of any aspect of this manuscript.

## Declaration of Competing Interest

None
